# The interaction between CaDUF642.01 and CaPME2 influences stem strength in *Capsicum annuum* L.

**DOI:** 10.1111/tpj.71074

**Published:** 2026-08-13

**Authors:** Yu Huang, Xinhui Li, Hongyu Zhou, Qingzhi Cui, Xiongze Dai, Fan Zhu, Xuexiao Zou, Junheng Lv, Lijun Ou, Minghua Deng, Huawu Zhu, Sha Yang

**Affiliations:** ^1^ Key Laboratory for Vegetable Biology of Hunan Province, Engineering Research Center for Horticultural Crop Germplasm Creation and New Variety Breeding Ministry of Education, College of Horticulture, Hunan Agricultural University Changsha China; ^2^ Key Laboratory of Vegetable Biology of Yunnan Province College of Landscape and Horticulture, Yunnan Agricultural University Kunming China; ^3^ College of Agriculture, Hunan Agricultural University Changsha China

**Keywords:** DUF642 gene family, *CaDUF642.01*, soluble pectin, water content, cell structure, stem strength

## Abstract

Stem strength is a key agronomic trait that influences planting adaptability and mechanical harvesting efficiency. Pepper is the most widely cultivated vegetable crop in China, and full mechanization of its production is crucial for enhancing quality and productivity. In the preliminary stage of this study, transcriptomic analysis was performed on pepper accessions exhibiting substantial differences in stem strength at various developmental stages. Through weighted gene co‐expression network analysis focused on genes involved in cell wall biosynthesis and transport, a candidate gene potentially regulating stem strength, designated CaDUF642.01, was identified. The CaDUF642.01 protein contains two DUF642 domains and localizes to the cell wall, plasma membrane, and nucleus. Silencing *CaDUF642.01* led to a 42.17% decrease in soluble pectin content, a 1.63% reduction in water content, a 43.18% shortening of internode length, and more compact cell arrangement. Furthermore, CaDUF642.01 was shown to directly interact with pectin methylesterase 2 (CaPME2), forming a protein complex that modulates pectin methylation levels via regulation of random PME demethylesterification, thereby influencing stem strength. Our results demonstrate that the CaDUF642.01‐CaPME2 complex attenuates stem strength by altering pectin composition through PME activity, which also increases stem water content and promotes looser parenchyma cell packing. This study reveals a novel regulatory mechanism of stem strength in pepper, providing a theoretical basis for manipulating plant architecture and breeding mechanization‐adapted pepper varieties.

## Significance Statement

Stem strength is a key agronomic trait affecting crop adaptability and mechanical harvesting efficiency. Unlike previous studies focused on lignin or cellulose, this work reveals that pectin methylesterase influences pepper stem strength by modulating pectin composition. We further identified the DUF642 gene family as a key regulator in this process, offering new insights into stem strength mechanisms and a theoretical foundation for breeding pepper varieties suitable for mechanized production.

## INTRODUCTION

The plant stem, as a crucial organ connecting roots to leaves, flowers, and fruits, plays an indispensable role in plant growth, development, and ecosystem function. Stems not only provide mechanical support, enabling plants to resist external forces like wind and rain without lodging, but also serve functions such as substance transport and nutrient storage. In agricultural production, stem strength directly relates to lodging resistance and final yield quality (Du et al., 2016, 2024). Morphological characteristics of plant stems, including plant height, stem diameter, stem wall thickness, internode length, and distribution, are primary factors affecting stem strength (Brulé et al., 2016). The cell wall plays an important role in supporting and defining plant cell shape; its composition and chemical structure change in response to environmental signals during plant development, allowing plant cells to control cell size by increasing their osmotic pressure and reducing cell wall extensibility (Cosgrove, 2005; Palin & Geitmann, 2012). The cell wall is a complex and dynamic structure primarily composed of cellulose, hemicellulose, pectin, and lignin, and can be modulated according to the needs of cell growth and development (Rui & Dinneny, 2020). Previous studies indicated that stem strength is mainly influenced by lignin or cellulose content in the stem. Low content leads to brittle stems in Arabidopsis (Jones et al., 2001), rice (Tanaka et al., 2003), buckwheat (Wang et al., 2015), soybean (Liu et al., 2016), herbaceous peony (Zhao et al., 2021), and pepper (Li, Fu, Hu, et al., 2024). Lodging‐resistant cultivars exhibit greater lignin or cellulose accumulation compared to lodging‐susceptible cultivars (Niu et al., 2021; Pesquet et al., 2025). Pectin is structurally and functionally the most complex polysaccharide in the plant cell wall. Pectin is a family of galacturonic acid‐rich polysaccharides including homogalacturonan (HG), rhamnogalacturonan I (RG‐I), and the substituted galacturonans rhamnogalacturonan II (RG‐II), and xylogalacturonan (XGA), possessing good extensibility and potentially playing a key role in cell wall remodeling (Mohnen, 2008; Zhang et al., 2021). A lower soluble/insoluble ratio (more insoluble pectin) typically signifies higher stem strength, and stem bending in fresh‐cut flowers corresponds to an increase in water‐soluble pectin (WSP) (Cheng et al., 2020). HG pectin is the primary mediator of changes in cell wall mechanical properties, thereby altering growth. PMEs play a key role in cell wall loosening and stiffening through HG demethylesterification (Levesque‐Tremblay et al., 2015; Peng et al., 2021; Wolf et al., 2009). Blockwise demethylesterification may lead to cell‐wall stiffening, whereas random demethylesterification may result in cell‐wall loosening (Hocq et al., 2017; Wang et al., 2022).

Pepper (*Capsicum annuum* L.) is an economically valuable and widely cultivated crop. Stem strength is an important agronomic trait affecting plant architecture. Most pepper cultivars have tall plant stature, multiple branches, and are prone to lodging, which is unfavorable for mechanical production. Rising labor costs make compact and shorter plant structures desirable for increasing planting density and improving crop yield. Pepper faces lodging risks during the fruit‐setting stage, which can lead to reduced yield and quality. Lodging increases labor and production costs and is a major limiting factor affecting crop yield, quality, and mechanical harvesting (Li et al., 2022; Li, Fu, Yang, et al., 2024). Suitable plant height also confers lodging resistance, making plants less susceptible to pathogens and suitable for mechanized management and harvest (Xing et al., 2024). With advancements in agricultural technology and increasing labor shortages, the transition from traditional to modern mechanized agriculture is imperative (Miao et al., 2024). In the preliminary stage of this study, transcriptomic analysis was performed on pepper accessions exhibiting substantial differences in stem strength at various developmental stages. Through weighted gene co‐expression network analysis (WGCNA) focused on genes associated with cell wall biosynthesis and transport, a member of the DUF642 gene family in pepper, designated CaDUF642.01, was identified as a candidate gene. The function of this gene in the stem was verified through silencing and overexpression of *CaDUF642.01*, the mechanism regulating stem strength was determined, and interacting proteins were identified to construct a gene regulatory network. This study reveals a novel regulatory mechanism for stem strength in pepper, providing a theoretical basis for plant architecture regulation and breeding pepper varieties suited for mechanization.

## RESULTS

### Identification and bioinformatic analysis of *CaDUF642.01*

**Figure 1:**
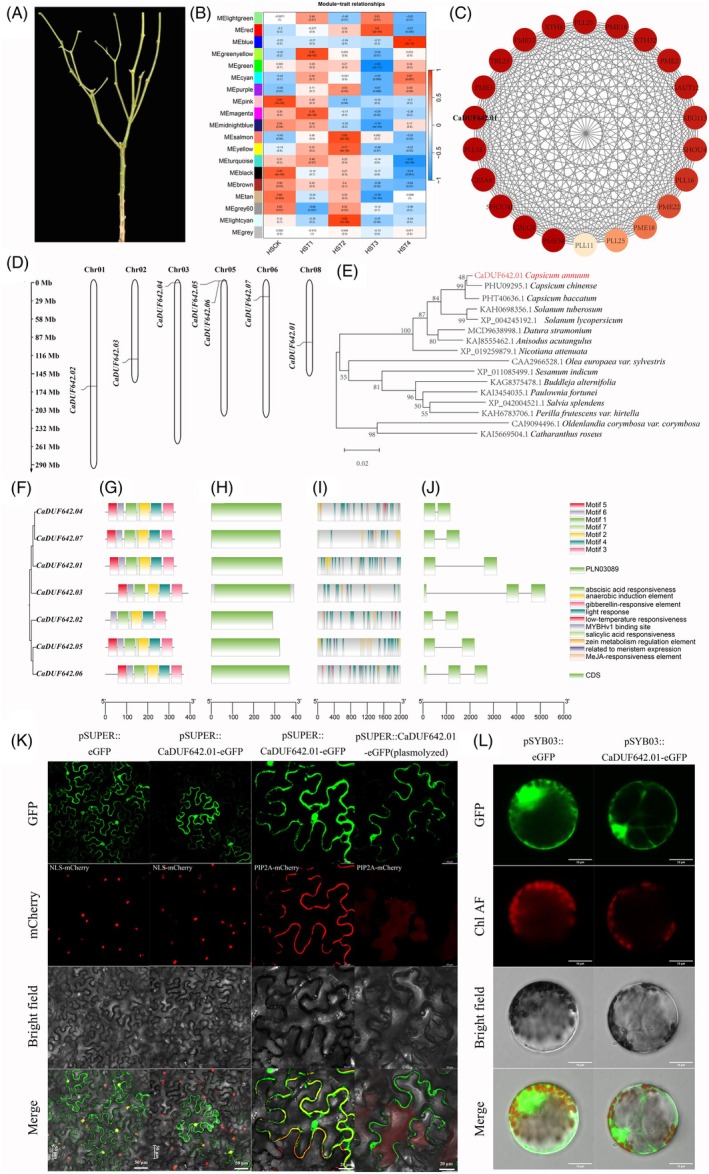
Identification of co‐expression modules associated with cell wall biosynthesis and remodeling genes and bioinformatic analysis of the CaDUF642 gene family. (A) The long‐stem pepper accession ‘A122’ used for analyzing temporal gene expression patterns related to stem cell wall components. (B) Module–trait association map. Each row corresponds to a module, and each column represents stem samples of ‘A122’ at different developmental stages. HSCK: the second internode above the cotyledon node at the four‐leaf and one‐heart stage; HST1, HST2, HST3: the same internode position on Days 5, 10, and 15 after treatment, respectively. (C) Network analysis of cell wall biosynthesis and transport genes within the MEturquoise module, highlighting CaDUF642.01. (D) Chromosomal distribution of CaDUF642 family genes. (E) Phylogenetic relationships among 16 species constructed using the amino acid sequence of CaDUF642.01. (F) Phylogenetic clustering of CaDUF642 proteins. (G) Motif patterns of CaDUF642 proteins. (H) Conserved domains within CaDUF642 proteins. (I) Cis‐acting elements in the 2000 bp upstream promoter regions of CaDUF642 genes. (J) Gene structure of CaDUF642. (K) Subcellular localization of the CaDUF642.01 protein in *Nicotiana benthamiana* leaves. Nuclear marker (NLS‐mCherry) or membrane marker (PIP2A‐mCherry) was separately co‐infiltrated with pSUPER::CaDUF642.01‐eGFP, and fluorescence signals were observed before and after plasmolysis treatment. (L) Subcellular localization of the CaDUF642.01 protein in *N. benthamiana* protoplasts. Scale bar = 10 μm.

To investigate the genetic basis of stem strength, the long‐stem pepper accession ‘A122’, which exhibits distinct stem strength phenotypes across different developmental stages, was selected as the experimental material for analyzing temporal gene expression patterns related to cell wall components (Figure 1A). WGCNA analysis was performed on transcriptome data from stem samples of ‘A122’ collected at the four‐leaf and one‐heart stage, and on the 5th, 10th, and 15th days thereafter, specifically from the second internode above the cotyledon node. A co‐expression network was subsequently generated (Figure 1B). Among these, the MEturquoise module exhibited the strongest correlation with the biosynthesis and modification of cell wall components (Figure 1B). This module encompassed key gene families involved in pectin biosynthesis and remodeling, including *PME* and *PLL*, as well as those responsible for cellulose and hemicellulose biosynthesis and remodeling, such as *CESA*, *CSL*, and *XTH*. Within the MEturquoise module, the transcriptional level of *CaDUF642.01* demonstrated a strong correlation with these cell wall synthesis‐ or remodeling‐related genes. Consequently, *CaDUF642.01* was identified as a potential regulatory gene involved in cell wall metabolism (Figure 1C). The DUF642 gene family members were distributed dispersedly across different chromosomes (Figure 1D), located on six chromosomes, with two *CaDUF642* genes on chromosome 5 physically very close to each other.

To elucidate the evolutionary relationships of the CaDUF642 gene among different species, a phylogenetic tree was constructed using the amino acid sequences of CaDUF642 from 16 species. The results showed that the 16 members were divided into three major clades. The first clade consisted of *C. annuum*, *Capsicum chinense*, *Capsicum baccatum*, *Solanum tuberosum*, *Solanum lycopersicum*, *Datura stramonium*, *Anisodus acutangulus*, and *Nicotiana attenuata*. The gene most closely related to pepper *CaDUF642.01* was from *C. chinense*. The second clade consisted of *Olea europaea* var. *sylvestris*, *Sesamum indicum*, *Buddleja alternifolia*, *Paulownia fortunei*, *Salvia splendens*, and *Perilla frutescens* var. *hirtella*. The third clade consisted of *Oldenlandia corymbosa* var. *corymbosa* and *Catharanthus roseus*, which were relatively distantly related and formed a small evolutionary branch with relatively low support (Figure 1E). A conserved motif search for the seven CaDUF642 proteins revealed five to six motifs per protein. Except for one gene lacking motif 1, all family members shared the same motif composition (Figure 1F–J). We extracted promoter sequences (2000 bp upstream of the start codon) of *CaDUF642* genes and identified their cis‐acting elements (Figure 1I). The exon number ranged from 2 to 3. Most *CaDUF642* genes lacked annotated 5′ or 3′ UTR information (Figure 1J). Transient expression of the CaDUF642.01‐GFP fusion protein in *Nicotiana benthamiana* leaf cells revealed that CaDUF642.01 was localized to the nucleus, plasma membrane, and cell wall, with a strong fluorescence signal in the cell wall and relatively weaker signal at the plasma membrane (Figure 1K). In addition, transient expression of the same fusion protein in *N. benthamiana* protoplasts showed that CaDUF642.01 was also distributed in the nucleus and plasma membrane, though the signal at the plasma membrane was weak (Figure 1L). Taken together, these subcellular localization results indicate that CaDUF642.01 is a multi‐compartment protein, predominantly enriched in the cell wall, followed by the nucleus and the plasma membrane, suggesting that it may be involved in biological processes such as cell wall metabolism or structural maintenance.

### Silencing *CaDUF642.01* affects pepper stem strength

**Figure 2:**
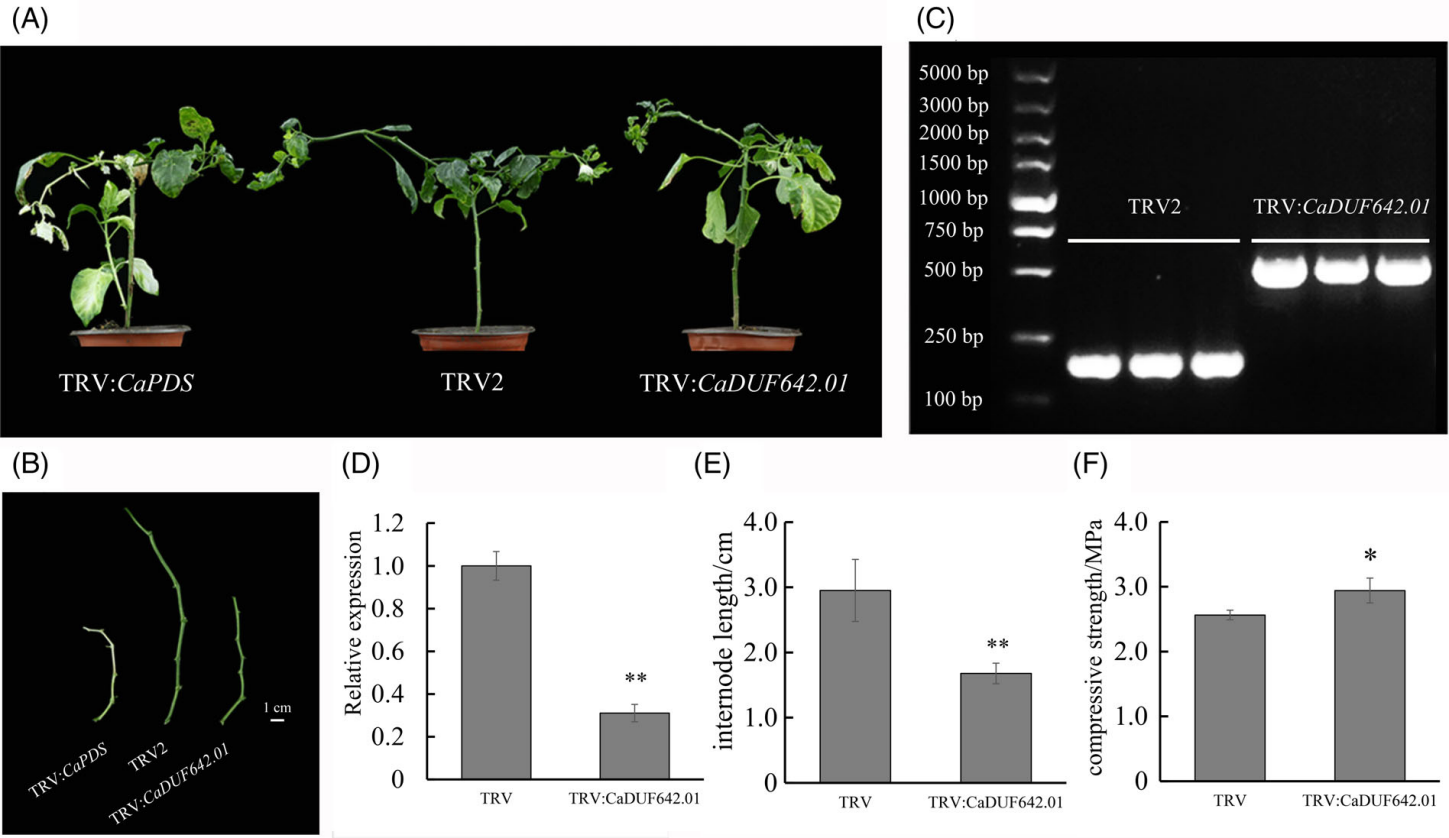
*CaDUF642.01* regulates pepper stem strength. (A) Plant morphology of *CaDUF642.01*‐silenced pepper plants. TRV2 and TRV:*CaPDS* vectors were used as controls. (B) Detail of stems. (C) PCR agarose gel identification. (D) Relative expression analysis of *CaDUF642.01* in TRV2 and TRV:*CaDUF642.01* materials. **, *P* < 0.01 (Student’s *t*‐test). (E) Internode length in TRV2 and TRV:*CaDUF642.01* materials. **, *P* < 0.01 (Student’s *t*‐test). (F) Stem compressive strength in TRV2 and TRV:*CaDUF642.01* materials. *, *P* < 0.05 (Student’s *t*‐test).

To confirm the function of *CaDUF642.01* in pepper, we used the VIGS system to silence its expression by constructing TRV:*CaDUF642.01*. Plants expressing the positive control vector TRV:*PDS* showed clear albino phenotypes, confirming successful VIGS silencing (Figure 2A). PCR of peppers inoculated with the empty TRV2 vector produced a 193‐bp target band (pTRV2), while PCR of peppers inoculated with TRV:*CaDUF642.01* produced a 553‐bp target band (pTRV2‐*CaDUF642.01*) (Figure 2C), confirming successful inoculation and expression of the recombinant vectors. The expression of *CaDUF642.01* in stems of TRV:*CaDUF642.01*‐infected peppers was significantly lower than in TRV2 empty vector‐infected stems (Figure 2D), confirming effective silencing of *CaDUF642.01*. Concurrently, the silenced stems showed significantly reduced internode length (Figure 2B,E) and significantly increased stem compressive strength (Figure 2F). Silencing *CaDUF642.01* expression significantly reduced internode length and increased stem compressive strength in pepper, indicating that CaDUF642.01 is involved in negatively regulating stem mechanical strength and internode elongation.

### Overexpression of *CaDUF642.01* affects tomato stem strength

**Figure 3:**
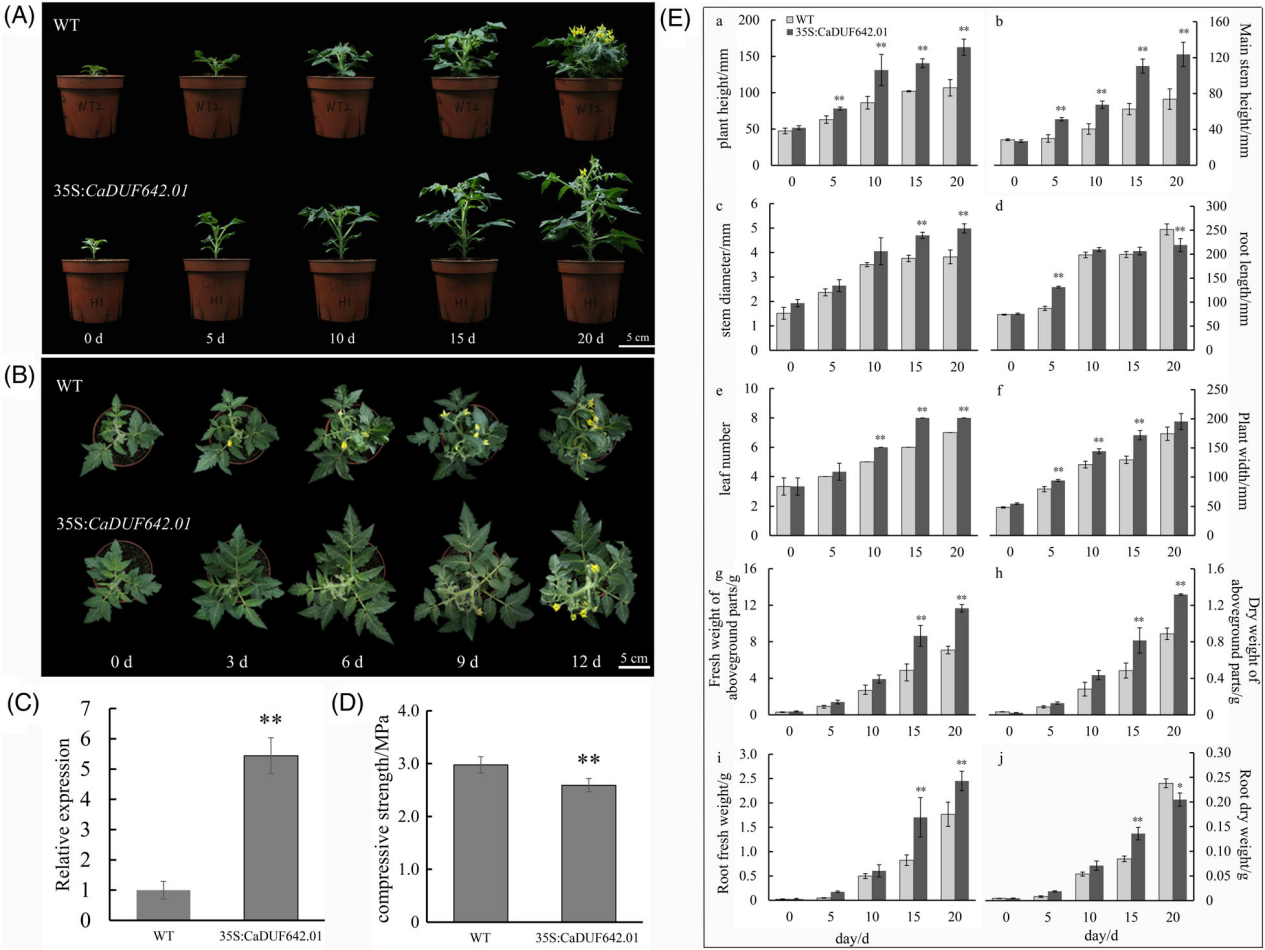
Identification of transgenic tomato *CaDUF642.01* and growth comparison with wild type (WT) at different days. (A) Phenotypic comparison of plant height and architecture between CaDUF642.01‐overexpressing tomato and WT at various developmental stages; (B) Flowering time variation between transgenic CaDUF642.01 and WT plants at the flowering stage; (C) RT‐qPCR analysis confirming significantly enhanced expression of 35S:CaDUF642.01; **, *P* < 0.01 (Student’s *t*‐test). (D) Comparison of stem compressive strength between 35S:CaDUF642.01 and WT plants; **, *P* < 0.01 (Student’s *t*‐test). (E) Agronomic trait data including (a, plant height; b, main stem height; c, stem diameter; d, root length; e, leaf number; f, plant spread; g, above‐ground fresh weight; h, above‐ground dry weight; i, root fresh weight; j, root dry weight). *, *P* < 0.05, **, *P* < 0.01 (Student’s *t*‐test).

Due to the low efficiency of genetic transformation and difficulty in plant regeneration in pepper, *CaDUF642.01* was heterologously overexpressed in tomato to obtain a sufficient number of stable transgenic lines for phenotypic analysis. We obtained stable transgenic overexpression lines. Phenotypic observation of growth and development in transgenic tomato plants revealed that stems were taller than wild‐type (WT). The transcript level was 5.44 times higher than in WT (Figure 3C). Stems of 35S:*CaDUF642.01* plants showed significantly lower compressive strength compared to WT (Figure 3D). Five plants each from the transgenic and WT lines were selected for observation and recording of agronomic traits. Growth from 0 to 20 days was statistically analyzed for transgenic 35S:*CaDUF642.01* and WT plants, revealing clear differences in growth rate. Phenotypic identification of transgenic lines by observing various agronomic traits at different days showed that at the 5‐day seedling stage, transgenic plants were significantly taller, with significantly elongated internodes, increased leaf number, and larger leaf size. Plant height, main stem height, and stem diameter were significantly increased (*P* < 0.01) (Figure 3A,E).

### *CaDUF642.01* influences stem strength by altering cell wall components

**Figure 4:**
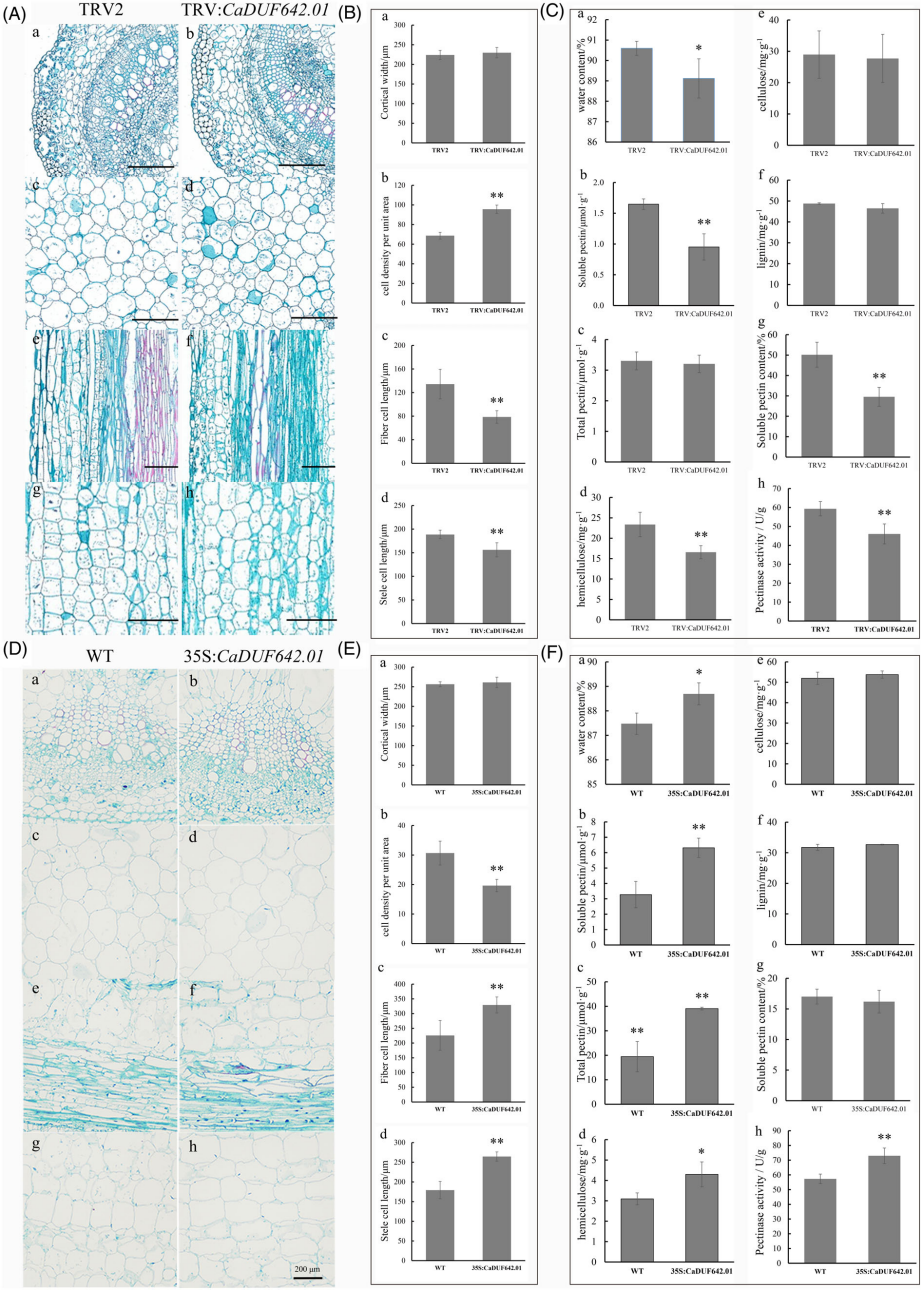
Cellular structure and cell wall components of pepper plants with silenced and overexpressed *CaDUF642.01* gene. (A) Histocytological images of stem tissue. (a) Cross‐section of cortical cells in TRV:CaDUF642.01 stem; (b) Cross‐section of cortical cells in TRV2 stem; (c) Cross‐section of stelar cells in TRV:CaDUF642.01 stem; (d) Cross‐section of stelar cells in TRV2 stem; (e) Longitudinal section of cortical cells in TRV:CaDUF642.01 stem; (f) Longitudinal section of cortical cells in TRV2 stem; (g) Longitudinal section of stelar cells in TRV:CaDUF642.01 stem; (h) Longitudinal section of stelar cells in TRV2 stem; Scale bar = 200 μm. (B) Cellular morphology parameters of TRV2 and TRV:CaDUF642.01: (a) cortex thickness; (b) number of stele cells per unit area; (c) fiber cell length; (d) stele cell length. **, *P* < 0.01 (Student’s *t*‐test). (C) Cell wall component contents of pepper plants with silenced *CaDUF642.01* gene and control TRV2 (a, water content; b, soluble pectin content; c, total pectin content; d, hemicellulose content; e, cellulose content; f, lignin content; g, proportion of soluble pectin in total pectin; h, pectinase activity). *, *P* < 0.05, **, *P* < 0.01 (Student’s *t*‐test). (D) Histocytological images of stem tissue. (a) Cross‐section of cortical cells in 35S:CaDUF642.01 stem; (b) Cross‐section of cortical cells in wild type (WT) stem; (c) Cross‐section of stelar cells in 35S:CaDUF642.01 stem; (d) Cross‐section of stelar cells in WT stem; (e) Longitudinal section of cortical cells in 35S:CaDUF642.01 stem; (f) Longitudinal section of cortical cells in WT stem; (g) Longitudinal section of stelar cells in 35S:CaDUF642.01 stem; (h) Longitudinal section of stelar cells in WT stem; Scale bar = 200 μm. (E) Cellular morphology parameters of WT and 35S:CaDUF642.01: (a) cortex thickness; (b) number of stele cells per unit area; (c) fiber cell length; (d) stele cell length. **, *P* < 0.01 (Student’s *t*‐test). (F) Cell wall component contents of tomato plants overexpressing *CaDUF642.01* and WT (a, water content; b, soluble pectin content; c, total pectin content; d, hemicellulose content; e, cellulose content; f, lignin content; g, proportion of soluble pectin in total pectin; h, pectinase activity). *, *P* < 0.05, **, *P* < 0.01 (Student’s *t*‐test).

Observation of cross‐sections of stem tissues from *CaDUF642.01*‐silenced pepper materials showed that in the cortex, cells were arranged more tightly, and the number of cells per unit area significantly increased. In the pith parenchyma cells, the xylem area in silenced materials was smaller than in WT materials (Figure 4Aa–d). Observation of longitudinal sections of stem cortex cells showed no significant difference in cortex thickness between TRV2 and TRV:*CaDUF642.01*, but TRV2 cortex cells were longer and larger, with increased cell size. The pith cell area in TRV:*CaDUF642.01* was larger, and the arrangement was looser (Figure 4Ba–d). In plants with silenced *CaDUF642.01* gene, the cell number and volume in the stem were reduced, and the cells were densely arranged, leading to a decrease in internode length. Analysis of stem components revealed that compared with TRV2, TRV:CaDUF642.01 exhibited significantly reduced water content, no significant changes in total pectin, lignin, or cellulose contents, significantly reduced soluble pectin and hemicellulose contents, a decreased proportion of soluble pectin, altered pectin composition, and significantly reduced pectinase activity (Figure 4Ca–h).

Observation of cross‐sections of stem tissues from transgenic tomato materials showed that in the cortex, the number of cells significantly increased, and cortex cell arrangement became looser. In the pith parenchyma cells, the xylem area in transgenic materials was larger than in WT materials (Figure 4Da–d). Observation of longitudinal sections of stem cortex cells showed little difference in cortex thickness between transgenic and WT materials; transgenic materials had larger pith cell area, while WT materials had smaller pith cells (Figure 4Ea–d). In transgenic plants overexpressing *CaDUF642.01*, the number and volume of stem cells increased. Analysis of tomato stem components revealed that, compared with the WT, although the fresh and dry weights of stems were significantly higher in *CaDUF642.01*‐overexpressing plants, the water content was also significantly increased, while no significant changes were observed in lignin or cellulose contents. The contents of total pectin, soluble pectin, and hemicellulose were significantly increased, and pectinase activity was also significantly elevated (Figure 4Fa–h). CaDUF642.01 promotes cell elongation and loose arrangement by modulating pectin metabolism (increasing soluble pectin and hemicellulose contents and enhancing pectinase activity), thereby positively regulating internode elongation and reducing mechanical strength. Conversely, silencing this gene results in tightly arranged and reduced cell volume, decreased soluble pectin and hemicellulose contents, and reduced pectinase activity, leading to shortened internodes and increased stem compressive strength. These findings indicate that CaDUF642.01 is a key negative regulator (with respect to strength) or positive regulator (with respect to internode elongation) affecting stem development and mechanical properties.

### *CaDUF642.01* affects pepper stem strength by interacting with CaPME2 protein

**Figure 5:**
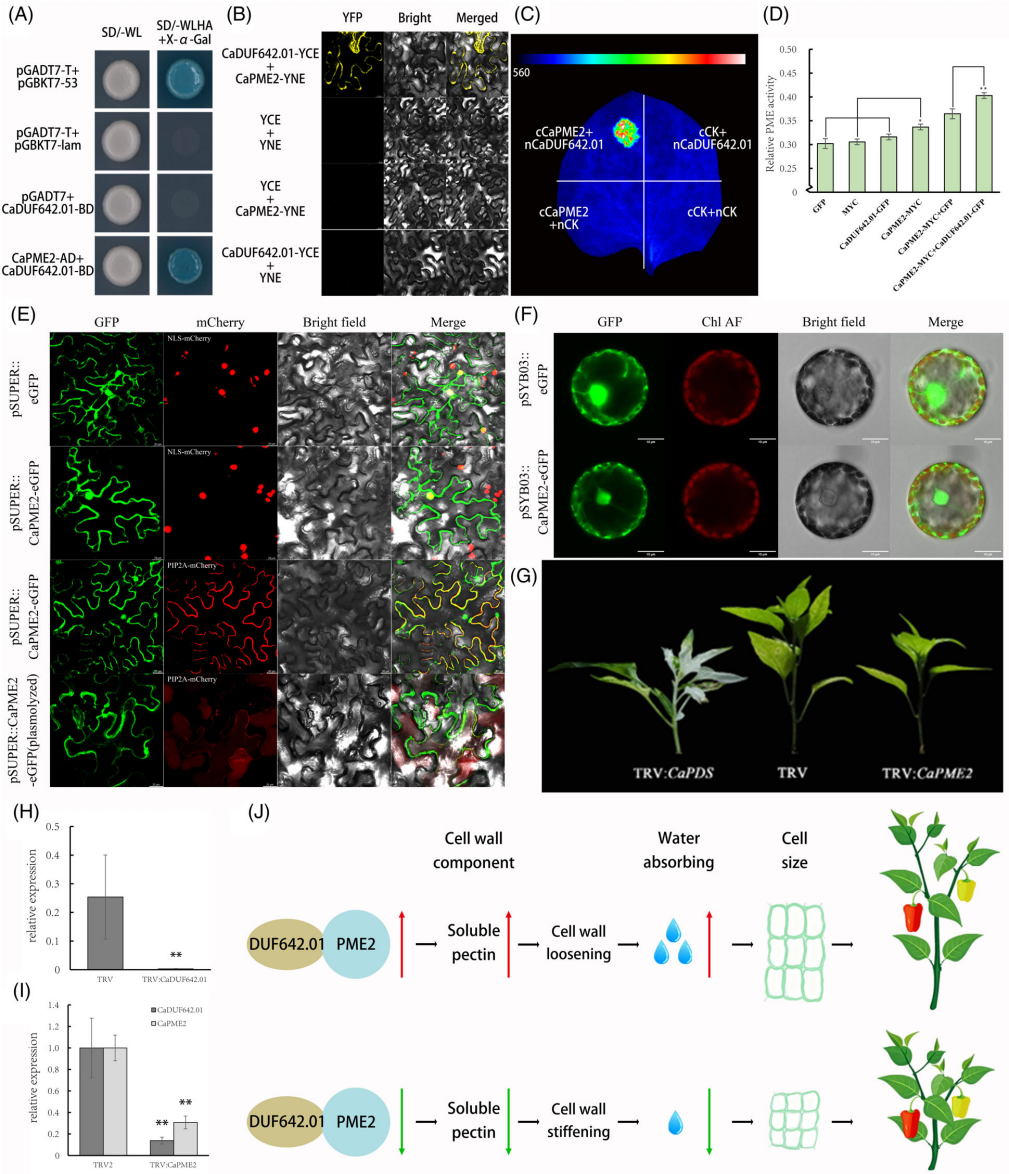
Interaction between CaDUF642.01 and CaPME2. (A) Yeast two‐hybrid (Y2H) assay showing the interaction between CaDUF642.01 and CaPME2; (B) Bimolecular fluorescence complementation (BiFC) assay demonstrating the interaction between CaDUF642.01 and CaPME2 at the plasma membrane of *Nicotiana benthamiana* leaf cells; (C) Luciferase complementation assay (LCA) detecting the *in vivo* interaction between CaDUF642.01 and CaPME2; (D) PME protein activity. CaDUF642.01 and CaPME proteins were purified by co‐immunoprecipitation (CO‐IP), and PME enzyme activity was measured *in vitro*. Negative controls were MYC and GFP proteins. CaDUF642.01‐GFP indicates CaDUF642.01 fused with a GFP tag. PME2‐MYC indicates the fusion of PME38 and PME2 with a MYC tag. *, *P* < 0.05, **, *P* < 0.01 (Student’s *t*‐test). (E) Subcellular localization of the CaPME2 protein in *N. benthamiana* leaves. Nuclear marker (NLS‐mCherry) or membrane marker (PIP2A‐mCherry) was separately co‐infiltrated with pSUPER::CaPME2‐eGFP, and fluorescence signals were observed before and after plasmolysis treatment. (F) Subcellular localization of CaPME2 protein in *N. benthamiana* protoplasts. Scale bar = 10 μm. (G) Expression level of *CaPME2* upon silencing of *CaDUF642.01*; (H) Phenotype of *CaPME2*‐silenced pepper plants; **, *P* < 0.01 (Student’s *t*‐test). (I) Relative expression levels of *CaDUF642.01* and *CaPME2* in *CaPME2*‐silenced plants; **, *P* < 0.01 (Student’s *t*‐test). (J) Schematic model.

After confirming that *CaDUF642.01* regulates pepper stem strength, we performed a Y2H yeast library screen to identify potential targets involved in the *CaDUF642.01*‐mediated pathway regulating stem strength and discovered an interaction between CaPME2 and CaDUF642.01 (Figure 5). Y2H results showed that CaPME2‐AD and CaDUF642.01‐BD did not show autoactivation. Y2H yeast cells co‐transformed with the CaDUF642.01‐BD and CaPME2‐AD combination grew well on SD‐Leu‐Trp‐His‐Ade selective medium and turned blue with X‐α‐Gal staining (Figure 5A). The bimolecular fluorescence complementation (BiFC) assay indicated that CaPME2 and CaDUF642.01 interact, likely at the cell membrane (Figure 5B). The interaction between CaDUF642.01 and CaPME2 was verified by the Split Firefly Luciferase Complementation (SFLC) assay, where fluorescence signals were detected when cLUC‐CaPME2 and nLUC‐CaDUF642.01 were co‐infiltrated into *N. benthamiana* leaves (Figure 5C). CaDUF642.01 and CaPME were transiently expressed in *N. benthamiana* leaves, and the encoded proteins were purified using co‐immunoprecipitation (CO‐IP). *In vitro* PME activity assays showed that protein extracts from plants transiently expressing CaDUF642.01‐GFP, GFP, or MYC alone had no effect on PME activity. However, extracts from leaves infiltrated with PME2‐MYC exhibited higher PME activity than those from leaves infiltrated with CaDUF642.01‐GFP or the two empty tag vectors. Importantly, when the CaDUF642.01‐GFP extract was added to the PME2‐MYC extract, PME activity increased. These results indicate that CaDUF642.01‐GFP promotes PME activity (Figure 5D). Subcellular localization assay of CaPME2 in *N. benthamiana* leaves indicated that the CaPME2 protein was expressed in the nucleus, plasma membrane, and cell wall, with high expression levels in the cell wall (Figure 5E). Subcellular localization analysis of CaPME2 in *N. benthamiana* protoplasts showed that CaPME2 is expressed in the plasma membrane and the nucleus (Figure 5F). Using the VIGS system, *CaPME2* expression in pepper was silenced by constructing TRV:*CaPME2*. Plants expressing the positive control vector TRV:*CaPDS* showed clear albino phenotypes, and *CaPME2* relative expression decreased, confirming successful VIGS silencing. Silenced stems showed significantly reduced internode length, indicating that the CaPME2 protein affects pepper stem strength by influencing internode length (Figure 5G,H). Silencing *CaDUF642.01* also reduced the relative expression level of *CaPME2* (Figure 5I).

## DISCUSSION

The Domain of Unknown Function 642 (DUF642) protein family is a seed plant‐specific family of cell wall‐associated proteins of unknown function, containing one to two highly conserved DUF642 domains (Vázquez‐Lobo et al., 2012). Reports on DUF642 family genes controlling plant stems are relatively scarce. DUF642 family members show differential expression in stems of *Medicago sativa* and hypocotyls of *Arabidopsis thaliana* (Abrahams et al., 1995; Minic et al., 2009). Arabidopsis *DUF642* gene mutants *bdx‐1* and *teb‐1* have longer hypocotyls compared to WT due to longer cell length, whereas overexpression lines produce shorter hypocotyls (Salazar‐Iribe et al., 2016). Expression of DUF642 family genes was detected in the primary root of Arabidopsis; all DUF642 genes were expressed in epidermal cells of the meristematic zone, and the *DGR2* mutant exhibited a short root phenotype (Gao et al., 2012). Overexpression of *AhDGR2* in Arabidopsis resulted in longer roots (Palmeros‐Suárez et al., 2017). Mutants like *bdx* and *teb* show altered hypocotyl phenotypes, suggesting DUF642 genes may be involved in regulating hypocotyl elongation during seedling development (Irshad et al., 2008). This study identified seven DUF642 gene family members in pepper through genome‐wide analysis. *CaDUF642.01* is located on chromosome 8, and its encoded protein contains two typical DUF642 domains (Figure 1). Further research found that silencing this gene resulted in a phenotype with significantly shorter stem internodes (Figure 2); overexpression in tomato showed significantly increased plant height and significantly elongated internodes (Figure 3).

Pectin is mainly present in the middle lamella and primary cell wall, enabling the adhesion of adjacent cells. Furthermore, pectin modification affects its gelling properties (Daher & Braybrook, 2015). Some studies show a highly significant positive correlation between pectin and stem strength, while stem strength in herbaceous peony is negatively correlated with pectin content at the mid‐development stage. Additionally, pectin content correlates with stem bending in other plants such as ramie, gerbera, and sugarcane (Huang et al., 2023). Reduced cell wall pectin content prevents stress‐induced root cell elongation in Arabidopsis (Liu et al., 2020). Pectin composition also influences stem strength; the solubility of pectin in the middle lamella and fiber cell walls decreases with stem maturation (Xu et al., 2019). Decreased content of WSP and chelator‐soluble pectin (CSP) increases cell wall porosity and extensibility (Fan et al., 2022). Stem bending in fresh‐cut flowers corresponds to an increase in WSP (Cheng et al., 2020). Our results show that silencing *CaDUF642.01* led to a 42.17% decrease in soluble pectin and altered pectin composition in pepper stems, along with a 1.63% reduction in stem water content. These changes were closely associated with increased cell packing density and enhanced stem strength. As a major component of the cell wall, the methylation status of pectin and the level of soluble pectin directly affect cell wall porosity and mechanical strength.

Changes in DUF642 gene expression patterns may be related to variations in pectin methylesterase activity or the degree of HG methylesterification during plant development in different cell types. Interactions between DUF642 proteins and polysaccharides/cell wall enzymes have been confirmed *in vitro*. PpDUF642 can interact with cellulose in the fruit cell wall to perform its function (Agustinelli et al., 2023). Proteins encoded by DUF642 in Arabidopsi*s* interact with cellulose‐specific enzymes (Esther & Alicia, 2012). Overexpression of the grape *VqDUF642* gene found higher PME activity in seedlings, stems, leaves, and fruits of transgenic Arabidopsis plants (Xie & Wang, 2016). Another study found that leaves of Arabidopsis plants overexpressing a *DUF642* gene showed reduced PME activity, while increased PME activity was detected in roots. Thus, DUF642 gene‐mediated regulation of pectin methylesterase contributes to the modulation of cell wall properties during plant growth (Cruz‐Valderrama et al., 2019). Our experiments confirmed that the CaDUF642.01 protein can directly interact with the CaPME2 protein to form a complex (Figure 5). Protein interaction network analysis suggests that CaDUF642.01 may act as a regulatory module, influencing the activity and stability of CaPME2 in the cell wall.

PME is a key enzyme catalyzing pectin demethylesterification; its activity directly regulates the pectin methylation level, thereby affecting cell wall structure and mechanical strength. PME regulates the mechanical and chemical properties of the plant cell wall through pectin demethylesterification, thereby promoting cell growth (Micheli, 2001). Blockwise demethylesterification leads to large stretches of demethylesterified GalA. These negatively charged chains can interact with cations, such as calcium ions, forming ‘egg‐box’ structures, described to favor cell wall rigidification, at least *in vitro*. At distinct pH values, the mode of action can shift to random demethylesterification. For both modes of action, PME activity generates low‐methylesterified HGs, which are substrates for pectin‐degrading enzymes such as PGs and pectin/pectate lyases (PLs/PNLs), forming oligogalacturonides that can lead to cell wall loosening (Hocq et al., 2017). Plants overexpressing certain PMEI genes exhibit delayed hypocotyl elongation (Palin & Geitmann, 2012; Wolf & Greiner, 2012). Transgenic potatoes overexpressing PME showed relatively faster elongation at early developmental stages (Pilling et al., 2000). Arabidopsis overexpressing *PME3* also produced longer roots and taller shoots, whereas the *pme3* knockout mutant showed the opposite phenotype (Hewezi et al., 2008). Primary cell wall demethylesterification mediated by Arabidopsis PME35 directly regulates the mechanical strength of supporting tissues (Hongo et al., 2012). This study showed that silencing the *CaPME2* gene resulted in a phenotype similar to silencing *CaDUF642.01*: shortened stem internodes and enhanced stem strength, further supporting the hypothesis that both genes act in the same genetic pathway (Figure 5). The CaDUF642.01‐CaPME2 complex likely affects cell expansion capacity and intercellular adhesion strength by regulating the physicochemical properties of pectin in the cell wall, ultimately modulating stem mechanical strength. This interaction may be an important mechanism for plants to adapt to mechanical stress and also provides a potential molecular target for crop breeding.

Based on the results of this study, we propose a working model where CaDUF642.01 influences stem strength by regulating pectin metabolism (Figure 5J). In this model, CaDUF642.01 interacts with CaPME2 to form a complex protein that regulates PME activity, affecting the methylation status of cell wall pectin, thereby altering pectin solubility and cross‐linking capacity, and ultimately regulating cell expansion capacity and stem mechanical strength. This regulatory pathway not only explains the molecular basis of stem strength and enriches the molecular regulatory theory of plant cell wall biosynthesis and stem development, but also provides a new perspective for understanding plant architecture development, offering important gene targets and a theoretical basis for molecular breeding of pepper varieties suitable for mechanical harvesting.

## MATERIALS AND METHODS

### Plant materials and growth conditions

Seeds uniform in size, plump, and free of impurities were selected and sown in plug trays. Germination and seedling cultivation were carried out in a growth chamber at 25°C with a 16 h light/8 h dark photoperiod and 65–75% air humidity. Seedlings were transplanted into nutrient pots with sufficient base water, observed, and cultured after a 3‐day acclimatization period for subsequent experiments.

### Subcellular localization, phylogenetic analysis, and bioinformatics

The individual fusion constructs pSUPER::CaDUF642.01‐eGFP, pSUPER::CaPME2‐eGFP, and the control construct (pSUPER::GFP) were co‐transformed into *N. benthamiana* leaves together with the membrane marker mCherry and the nuclear marker mCherry. The transformed tobacco plants were cultured for 48 h and then imaged using a confocal laser scanning microscope (Leica STELLARIS DMi8, Leica Microsystems, Wetzlar, Germany). After observation, the leaf tissues were immersed in a 50% sucrose solution for 20 min, and then the co‐localization of fluorescence signals was examined again under the same confocal microscope. For protoplast transformation, the constructs pSYB03::CaDUF642.01‐eGFP or pSYB03::CaPME2‐eGFP were co‐transformed with mCherry into *N. benthamiana* mesophyll protoplasts. An aliquot of 100 μl protoplast suspension was mixed with 10 μl plasmid DNA and 110 μl PEG4000, gently vortexed, and incubated at room temperature for 10–15 min. The reaction was terminated by adding W5 solution, followed by centrifugation and collection of protoplasts. After washing, the protoplasts were resuspended in W5 and incubated in darkness at 25°C for 18–24 h. Prior to observation, approximately 100 μl of the suspension was retained and examined under a confocal laser scanning microscope. Based on the cloned *CaDUF642.01* gene sequence from pepper, the ProtParam online tool (https://web.expasy.org/protparam/) was used for analyzing the physicochemical properties of the amino acids. MEGA6.0 software was used for amino acid sequence alignment and phylogenetic tree construction. DNAMAN software was used for amino acid sequence analysis. The NCBI CDD database was used to predict protein conserved domains. TBtools software was used for the analysis and processing of various bioinformatics data.

### Virus‐induced gene silencing (VIGS)

To construct the VIGS vectors, the full‐length sequences of *CaDUF642.01* and *CaPME2* were submitted to the VIGS tool on the Sol Genomics website (https://vigs.solgenomics.net/) to identify the most effective regions for silencing. Referring to previous VIGS methods (Zhou et al., 2021), the pTRV2 vector was digested with EcoRI and BamHI. After homologous recombination, PCR identification and sequencing of monoclonal colonies were performed using TRV2 F/R primer pairs. Plants were inoculated via leaf infiltration using an infiltration buffer (10 mM MgCl_2_, 10 mM MES, 200 μM AS). TRV2 empty vector and vector containing the phytoene desaturase (*PDS*) gene were used as controls. After inoculation, plants were kept in darkness for 24 h, then transferred to a controlled environment chamber with a 16 h light (200 μmol m^−2^ sec^−1^, 22°C ± 2°C)/8 h dark (20°C ± 2°C) photocycle and 70% relative humidity. Photobleaching in leaves of positive control plants indicated successful inoculation. PCR using TRV virus primers (TRV2 F/R) on new leaf tissue identified positive VIGS plants. Silencing efficiency in positive plants was evaluated by quantitative real‐time PCR, and plant phenotypes were observed.

### RNA extraction, reverse transcription, and quantitative PCR

Total RNA was extracted using the SteadyPure Plant RNA Extraction Kit, and first‐strand cDNA was synthesized using the HiScript II First Strand cDNA Synthesis Kit (Vazyme, Nanjing, Jiangsu, China). Quantitative real‐time PCR was performed on a real‐time PCR instrument using ChamQ Universal SYBR qPCR Master Mix (Vazyme) according to the manufacturer's instructions. The housekeeping gene *β‐actin* was used as the internal reference. All primers used are listed in Table S1.

### Plasmid construction and genetic transformation of transgenic tomato plants

Tomato seeds were sterilized and sown on 1/2 MS medium, dark‐cultured until germination, then transferred to light conditions for 6–8 days. Sterile cotyledons were excised and pre‐cultured for 2 days. *Agrobacterium tumefaciens* was activated on medium containing 50 mg L^−1^ kanamycin. Cotyledons were infected with *Agrobacterium*, then co‐cultured in the dark for 2 days. Explants were transferred to selection medium and subcultured every 2 weeks until green shoot spots appeared. Resistant shoots were transferred to rooting medium and cultured under light for 2–3 weeks until roots formed. Leaves from regenerated plants were collected, genomic DNA was extracted using the CTAB method, and PCR detection was performed using specific primers for the selectable marker gene.

### Stem hardness measurement and cell wall component analysis

At the first flowering stage when stem strength became relatively stable, the second internode from the bottom was selected, and its compressive strength was measured using a stem hardness tester (TP‐YYD‐1, China). The contents of various cell wall components were determined using kits from Solarbio (Solarbio, Beijing, China. https://www.solarbio.com/). Kit catalog numbers were: Total Pectin Content Assay Kit (BC1405), Soluble Pectin Content Assay Kit (BC4125), Cellulose Content Assay Kit (BC4285), Hemicellulose Content Assay Kit (BC4445), Lignin Content Assay Kit (BC4205), Pectinase Activity Assay Kit (BC2635). Measurements and calculations were performed according to the kit instructions.

### Cell structure observation

Stem tissues were observed using paraffin sectioning with safranin and fast green staining. Tissues were fixed in FAA fixative, dehydrated through an ethanol series, and embedded in paraffin. Sections were stained with safranin solution, destained in 50, 70, and 80% ethanol, stained with fast green solution, dehydrated again with ethanol, cleared in xylene for 5 min, and mounted with neutral balsam. Sections were examined under a microscope (Nikon Eclipse E100), and images were captured and analyzed.

### Yeast two‐hybrid assay (Y2H)

The cDNAs of *CaDUF642.01* and *CaPME2* were cloned into the Y2H prey vector pGADT7 and bait vector pGBKT7, respectively. After confirming the absence of autoactivation or toxicity for the bait constructs, GAL4‐based Y2H screening was performed. Specified bait and prey constructs were co‐transformed into the yeast strain Y2H Gold, and co‐transformed yeast clones were cultured on selective media.

### Luciferase complementation assay (LCA)

The coding sequences of *CaDUF642.01* and *CaPME2* were cloned into pCAMBIA1300‐nLUC and pCAMBIA1300‐cLUC vectors, respectively, and transformed into *A. tumefaciens* strain GV3101. *Agrobacterium* strains containing the specified plasmids and the p19 plasmid (to suppress gene silencing) were co‐infiltrated into leaves of *N. benthamiana* for transient expression. After 48 h, d‐luciferin potassium salt was sprayed onto the abaxial side of the leaves, and luminescence images were captured using a plant live imaging system (DYNAPLANT, China).

### Bimolecular fluorescence complementation assay

The full‐length coding sequence of *CaDUF642.01* was ligated into the pSPYNE‐35S vector, and the full‐length coding sequence of *CaPME2* was ligated into the pSPYCE‐35S vector. The resulting plasmids were transformed into *A. tumefaciens* strain GV3101. The *Agrobacterium* cultures containing CaDUF642.01‐YNE, CaPME2‐YCE, and the p19 plasmid (for suppressing gene silencing) were co‐cultured in infiltration buffer at equal concentrations for 3 h. The bacterial mixture was then infiltrated into leaves of *N. benthamiana* plants. Fluorescence signals were examined using a confocal laser scanning microscope 2–3 days post‐infiltration.

### Determination of PME enzyme activity

The activity of PME enzyme in pepper stems was measured according to published methods (Hongo et al., 2012; Huang et al., 2017). The activity of the expressed PME proteins was also examined. The constructs CaDUF642.01‐GFP and PME2‐MYC were generated and transformed into *Agrobacterium* for transient expression. Empty GFP and MYC vectors were used as controls. The extracted proteins were immunoprecipitated using MYC beads to enrich the PME proteins. At least three replicates were used for each sample to improve data accuracy.

## AUTHOR CONTRIBUTIONS

YH, SY, and LO planned and designed the research. YH, XL, and H Zhou performed most of the experiments. QC and JL analyzed the data. YH and H Zhu wrote the manuscript. XZ, XD, FZ, and MD provided technical assistance. YH, XL, and H Zhou contributed equally to this work.

## CONFLICT OF INTEREST

The authors declare that there are no conflicts of interest.

## Supporting information


**Table S1.** Primer sequence.

## Data Availability

The authors confirm that the data supporting the findings of this study are available within the article or its Supporting Information.
